# The work to swing limbs in humans versus chimpanzees and its relation to the metabolic cost of walking

**DOI:** 10.1038/s41598-024-59171-8

**Published:** 2024-04-18

**Authors:** Francesco Luciano, Luca Ruggiero, Alberto E. Minetti, Gaspare Pavei

**Affiliations:** 1 Department of Pathophysiology and Transplantation, University of Milan, 20133 Milan, Italy; 2 Human Performance Research Centre, Department of Sports Science, University of Konstanz, Konstanz, Germany

**Keywords:** Biomechanics, Physiology

## Abstract

Compared to their closest ape relatives, humans walk bipedally with lower metabolic cost (C) and less mechanical work to move their body center of mass (external mechanical work, W_EXT_). However, differences in W_EXT_ are not large enough to explain the observed lower C: humans may also do less work to move limbs relative to their body center of mass (internal kinetic mechanical work, W_INT,k_). From published data, we estimated differences in W_INT,k_, total mechanical work (W_TOT_), and efficiency between humans and chimpanzees walking bipedally. Estimated W_INT,k_ is ~ 60% lower in humans due to changes in limb mass distribution, lower stride frequency and duty factor. When summing W_INT,k_ to W_EXT_, between-species differences in efficiency are smaller than those in C; variations in W_TOT_ correlate with between-species, but not within-species, differences in C. These results partially support the hypothesis that the low cost of human walking is due to the concerted low W_INT,k_ and W_EXT_.

## Introduction

Humans walk with lower metabolic energy demands than their closest ape relatives^1–3^. This may have enabled them to economically forage in environments with low food density and has been pivotal for their expansion and prosperity^1,4,5^. To understand how such economical locomotion is achieved, researchers have compared humans to chimpanzees, since they are phylogenetically close to humans and facultative bipeds when free-ranging^6–10^: humans expend less than half metabolic energy than chimpanzees during bipedal locomotion, and such a difference correlates with active limb muscle volume estimated through inverse dynamics^1–3,5,11^. Coherently, humans walk with more favourable pendular mechanics of their body center of mass and do ~ 50% less work to lift and accelerate it compared with chimpanzees (external mechanical work, W_EXT_)^6,12^. Differences in body center of mass mechanics may be driven by anatomical factors, such as longer hindlimbs in humans^13^, narrower pelvis with a shorter and more dorsally projecting ischium^14^, greater bicondylar valgus knee angle^6,15^, a more adducted hallux and stiffer midfoot^16,17^, the latter aspects favoring the ability to walk with a heel-to-toe rolling pattern^18^ and push-off mechanics^17^. Recently, O’Neill and colleagues^19^ have also shown that the summed dimensionless joint work at hip, knee, and ankle joints is ~ 25% lower in humans than chimpanzees, and ~ 45% lower when elastic energy storage is accounted for.

However, do the observed differences in walking mechanics fully explain reductions in metabolic demands? In humans, W_EXT_ is 50–70% of *total mechanical work* (W_TOT_)^20^ so a 50% lower W_EXT_, without changes in efficiency, would lower metabolic demands by no more than 35%. W_TOT_ also includes the work done to swing limbs with respect to the body center of mass (*internal kinetic mechanical work, W*_*INT,k*_)^21,22^, which may be sensibly lower in humans than in chimpanzees based on several observations. Humans have a two-fold lower moment of inertia of the upper limb^23,24^, which lowers the work required to swing it^19,25^. Moreover, Human lower limb is longer than chimpanzees’ hindlimb^23,24,26^. This increases the moment of inertia but decreases the number of acceleration-deceleration cycles for a given walking distance^27^: at matched speeds, humans walk with lower stride frequencies than chimpanzees^2,28,29^. Finally, humans may also walk with a lower duty factor^2,28^—the fraction of the stride period in which a limb contacts the ground—which reduces limb acceleration during swing. Although well-characterized in humans, W_INT,k_ is unknown for chimpanzees walking bipedally. Knowing it would allow a comparison between the two species and an assessment of differences in W_TOT_ and locomotor efficiency, the ratio of mechanical work to metabolic cost^21^. In the present work, we analyze literature data on bipedal walking in the two species and assess the following hypotheses: (i) W_INT,k_ is substantially lower in humans than in chimpanzees; (ii) once W_INT,k_ is accounted for, interspecies differences in W_TOT_ are approximately proportional to differences in metabolic demands.

## Materials and methods

### Data sources

This work draws on published data on bipedal walking for chimpanzees^2,6^ and humans^29^. All such data are available in text, tables, figures, and supplementary materials of the cited papers except for duty factor data from Pavei et al.^29^, which were shared by the authors. The following sections show how mechanical and metabolic variables were estimated from them. Table 1 summarizes the demographic and biometric characteristics of the study participants.

**Table 1:** Demographic and biometric characteristics of the study participants.

Source	Species	N	Sex	Age (years)		Body mass (kg)		Lower limb or hindlimb length (m)	
				Mean	SD	Mean	SD	Mean	SD
Pontzer et al.^2^	Chimpanzees	5	F: 3 M: 2	19	11	59.9	19.5	0.46	0.05
Demes et al.^6^	Chimpanzees	3	Not specified	6	0	28.7	6.4	0.38	0.03
Pavei et al.^29^	Humans	13	F: 7 M: 6	23	3	62.4	10.0	0.90	0.03

For Demes et al.^6^, no information could be retrieved about sex.

### Internal kinetic mechanical work

Experimental measurements of W_INT,k_ are unavailable for chimpanzees. However, in legged animals, W_INT,k_ (J kg^−1^ m^−1^) can be modeled as^28^:

1 $${W}_{INT,k}=SF v \left(1+{\left(\frac{d}{1-d}\right)}^{2}\right)q$$

where *SF* is the stride frequency (Hz), *v* is the average progression speed (m s^−1^), *d* is the duty factor, and *q* is a dimensionless term that depends on the inertial properties of the limbs:

2 $$q=\frac{{\pi }^{2}}{4}\left[\left({a}^{2}+{\gamma }^{2}\right)\left({m}^{\prime}_{L}+{b}^{2}{m}^{\prime}_{U}\right)\right]$$

where *a* and γ are the average proximal distance and gyration radius of the lower limb center of mass as a fraction of limb length, *b* is the upper limb length as a fraction of the lower one, and *m’*_*L*_ and *m’*_*U*_ are the masses as a fraction of body mass of the lower and upper limbs, respectively^28^. This equation neglects differences in relative gyration radius between upper and lower limbs, which may be inappropriate when comparing W_INT,k_ between species since the proportional mass distribution between fore- and hindlimbs differs between humans and chimpanzees^24,26,30,31^. A more general version of Eqs. (1) and (2) can be written from the original formulation by Minetti and Saibene^32^:

3 $${\dot{W}}_{INT, k}=SF {v}^{2}\frac{{\pi }^{2}}{2}[{a}^{2}\left({m}_{L}+{b}^{2}{m}_{U}\right)+\left({m}_{L}{\gamma }_{L}^{2}+{m}_{U}{b}^{2}{\gamma }_{U}^{2}\right)]$$

where Ẇ_INT,k_ is the mechanical internal power, and γ_*L*_ and γ_*U*_ are the gyration radii of the lower and upper limbs as a fraction of the respective limb length. To account for the duty factor, *v*^*2*^ can be written as^28^:

4 $${v}^{2}=\frac{1}{2}{v}_{ST}^{2}+\frac{1}{2}{v}_{SW}^{2}$$

where *v*_*ST*_ is the progression speed term, and *v*_*SW*_ is the term for the limb speed relative to the body center of mass. The relation between *v*_*SW*_ and the duty factor (d) is given by:

5 $${v}_{SW}={v}_{ST}\left(\frac{d}{1-d}\right)$$

Combining (4) and (5) yields:

6 $${v}^{2}=\frac{1}{2}{v}_{ST}^{2}\left(1+{\left(\frac{d}{1-d}\right)}^{2}\right)$$

Therefore, Ẇ_INT,k_ is:

7 $${\dot{W}}_{INT, k}=SF {v}_{ST}^{2}\left(1+{\left(\frac{d}{1-d}\right)}^{2}\right)\frac{{\pi }^{2}}{4}[{a}^{2}\left({m}_{L}+{b}^{2}{m}_{U}\right)+\left({m}_{L}{\gamma }_{L}^{2}+{m}_{U}{b}^{2}{\gamma }_{U}^{2}\right)]$$

Defining *m’*_*L*_ and *m’*_*U*_ as the fractional masses of the upper and lower limbs, and *m* as the total body mass:

8 $${\dot{W}}_{INT,k}=m SF {v}_{ST}^{2}\left(1+{\left(\frac{d}{1-d}\right)}^{2}\right)\frac{{\pi }^{2}}{4}\left[{a}^{2}\left({{m}^{\prime}}_{L}+{b}^{2}{{m}^{\prime}}_{U}\right)+\left({{m}^{\prime}}_{L }{\gamma }_{L}^{2}+{{m}^{\prime}}_{U}{b}^{2}{\gamma }_{U}^{2}\right)\right]$$

Converting from mechanical power to the mechanical work performed to move a unit body mass per unit distance (J kg^−1^ m^−1^):

9 $${W}_{INT,k}=SF {v}_{ST}\left(1+{\left(\frac{d}{1-d}\right)}^{2}\right)\frac{{\pi }^{2}}{4}\left[{a}^{2}\left({{m}^{\prime}}_{L}+{b}^{2}{{m}^{\prime}}_{U}\right)+\left({{m}^{\prime}}_{L }{\gamma }_{L}^{2}+{{m}^{\prime}}_{U}{b}^{2}{\gamma }_{U}^{2}\right)\right]$$

This equation only differs from the equation presented in the work of Minetti^28^ in that it does not assume equal relative gyration radii for the upper and lower limbs. The term *q’* can be defined here as:

10 $${q}^{\prime}=\frac{{\pi }^{2}}{4}\left[{a}^{2}\left({{m}^{\prime}}_{L}+{b}^{2}{{m}^{\prime}}_{U}\right)+\left({{m}^{\prime}}_{L }{\gamma }_{L}^{2}+{{m}^{\prime}}_{U}{b}^{2}{\gamma }_{U}^{2}\right)\right]$$

For which q is a special case when a unique radius of gyration relative to limb length (γ) is assumed for the upper and lower limbs (γ_*L*_ = γ_*U*_ = γ*)*. Hence:

11 $${W}_{INT,k}=SF {v}_{ST}\left(1+{\left(\frac{d}{1-d}\right)}^{2}\right){q}^{\prime}$$

This allowed estimating W_INT,k_ for chimpanzees based on spatiotemporal data from Pontzer et al.^2^; for humans, W_INT,k_ values were taken from Pavei et al.^29^. This model assumes extended limbs but can be expanded to account for the bent-hip, bent-knee features of chimpanzees walking; the validity of such mechanical work estimates is discussed in Supplementary Material S1.

In addition to W_INT,k_, work is done to overcome joint frictions during locomotion *(internal frictional mechanical work*, W_INT,f_; J kg^−1^ m^−1^)^33^; this term is not estimated here for chimpanzees because experimental data on limb damping are lacking (Supplementary Material S2).

### External mechanical work and total mechanical work

For humans, external mechanical work (W_EXT_) increases with walking speed^12,20,29^; however, for chimpanzees, such a relationship is less clear. Here W_EXT_ data for chimpanzees walking bipedally were taken from Demes et al.^6^ and fitted with zero, first- and second-order mixed effect models in the forms:

12 $${W}_{EXT}={\upbeta }_{0}+b \left(1 | participant\right)+ \epsilon$$

13 $${W}_{EXT}={\upbeta }_{0}+{\beta }_{1} speed+b \left(1 | participant\right)+ \epsilon$$

14 $${W}_{EXT}={\upbeta }_{0}+{\beta }_{1} speed+{\beta }_{2} spee{d}^{2}+b \left(1 | participant\right)+ \epsilon$$

where β and b are the fixed and random effect coefficients, respectively. The Akaike Information Criterion (AIC) was calculated, and the model with the lowest AIC was chosen. A zero-order model had the lowest AIC (Supplementary Material S3), so all the analyses in the present work used a speed-independent value of 0.55 ± 0.18 J kg^−1^ m^−1^, equal to the mean W_EXT_ reported by Demes and colleagues^6^. All these analyses were done with R 3.6.2, R Studio 1.2, and lme4^34–36^. W_TOT_ was then calculated as the sum of W_INT,k_ and W_EXT_, and its standard deviation as^37^:

15 $${SD}_{{W}_{TOT}}= \sqrt{{SD}_{{W}_{INT,k}}^{2}+{SD}_{{W}_{EXT}}^{2}}$$

where SD_WINT,k_ and *SD*_*WEXT*_ are the standard deviations for W_INT,k_ and W_EXT_, respectively. For humans, experimental values for W_INT,k_, W_EXT_ and W_TOT_ were taken from Pavei et al.^29^.

### Stride frequency and duty factor

**Figure 1:**
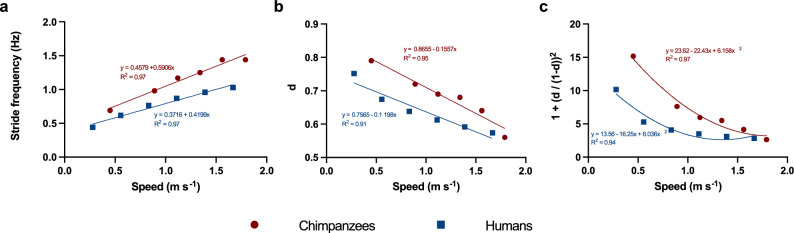
Spatiotemporal parameters. Stride frequency, duty factor (d) and the term 1 + (d/(1 − d))^2^ from Eq. (11) are plotted for chimpanzees (red circles; data from Pontzer et al.^2^) and humans (blue squares; data from Pavei et al.^29^). Species-specific linear and polynomial regression equations are shown, together with their coefficient of determination (R^2^).

For each species, stride frequency and duty factor values from Pavei et al.^29^ and Pontzer et al.^2^ were regressed over speed (Fig. 1). Then, percent variations were calculated from regression equations at the minimum (0.45 m s^−1^) and maximum (1.67 m s^−1^) common speeds between the two datasets and reported in Table 2. The uncertainties for SF and d were quantified by their standard deviations SD_SF_ and SD_d_, and propagated as:

16 $${SD}_{{W}_{INT,k}}= \sqrt{{\left(\frac{\partial {W}_{INT,k}}{\partial SF}\cdot {SD}_{SF}\right)}^{2}+{\left(\frac{\partial {W}_{INT,k}}{\partial d}\cdot {SD}_{d}\right)}^{2}}$$

to estimate how they impacted SD_WINT,k_^37^. Of note, duty factor values were taken from Pontzer et al.^2^, but O’Neill and colleagues^38^ reported similar duty factors between three chimpanzees and three speed-matched humans. Despite this, duty factor values from the former study were chosen due to the larger number of chimpanzee participants and a wider range of walking speeds. In instances of smaller differences in duty factor, the resulting differences in W_INT,k_ would be smaller but still be present, as indicated by error propagation and Table 2.

**Table 2:** Determinants of W_INT,k_.

Parameter	Description	Chimpanzees	Humans	% difference
Inertial parameters				
a	Proximal distance of the lower limb center of mass as a fraction of lower limb length	0.336	0.280	− 17%
b	Upper limb length as a fraction of lower limb length	1.032	0.585	− 43%
m’_U_	Upper limb mass as a fraction of body mass	0.084	0.047	− 44%
m’_L_	Lower limb mass as a fraction of body mass	0.122	0.203	+ 67%
γ_U_	Radius of gyration of the upper limb as a fraction of limb length	0.273	0.281	+ 3%
γ_L_	Radius of gyration of the lower limb as a fraction of limb length	0.268	0.259	− 3%
q’	Inertial factor, given by $\frac{{\pi }^{2}}{4}\left[{a}^{2}\left({{m}^{\mathrm{^{\prime}}}}_{L}+{b}^{2}{{m}^{\mathrm{^{\prime}}}}_{U}\right)+\left({{m}^{\mathrm{^{\prime}}}}_{L }{g}_{L}^{2}+{{m}^{\mathrm{^{\prime}}}}_{U}{b}^{2}{g}_{U}^{2}\right)\right]$	0.096	0.081	− 16%
Spatiotemporal parameters				
SF	Stride frequency (Hz)	[0.72; 1.44]	[0.56; 1.07]	[− 26%; − 22%]
d	Duty factor	[0.61; 0.80]	[0.56; 0.70]	[− 13%; − 8%]
$1+{\left(\frac{d}{1-d}\right)}^{2}$	Function relating duty factor to W_INT,k_ in Eq. (11)	[3.34; 14.77]	[3.26; 7.47]	[− 49%; − 2%]

Human parameters were calculated from De Leva et al.^23^ and Pavei et al.^29^, mean of females and males. Parameters for chimpanzees were calculated from Druelle et al.^39^ and Pontzer et al.^2^, mean of females and males. For spatiotemporal parameters, brackets report the minimum and maximum values and percent variations in the common speed range (0.45–1.67 m s^−1^). % difference is calculated with respect to chimpanzee values.

### Metabolic cost and efficiency

To calculate efficiency, metabolic demands must be expressed in the same units as mechanical ones. Pontzer et al.^2^ measured the oxygen uptake of five chimpanzees walking bipedally on a treadmill at various speeds. From these data, metabolic cost C (J kg^−1^ m^−1^) can be calculated as^40,41^:

17 $$C=\frac{\left(\dot{V}{O}_{2ss}-\dot{V}{O}_{2rest}\right) Eq{O}_{2}}{v m}$$

where *V̇O*_*2ss*_ and *V̇O*_*2rest*_ are the oxygen uptake during steady-state locomotion and at rest, respectively, *m* is the body mass (kg), and *EqO*_*2*_ is the number of joules released during the combustion of one milliliter of oxygen. *EqO*_*2*_ spans from 19.62 to 21.13 J mLO_2_^–1^^42^, and here a mean value of 20.9 J per mLO_2_ is assumed. Efficiency is W_TOT_ C^−1^^21^; therefore, its standard deviation is given by^37^:

18 $${SD}_{efficiency}= \sqrt{\frac{{W}_{TOT}^{2}{SD}_{C}^{2}+{SD}_{{W}_{TOT}}^{2}{C}^{2}}{{C}^{4}}}$$

where *SD*_*C*_ is the sample standard deviation for C. For humans, Pavei and colleagues^29^ provide experimental measurements of C and efficiency. Each outcome variable was regressed over speed; due to the small sample size and the unsuitability of null hypothesis testing for such a study design, only regression parameters were reported together with their coefficient of determination (R^2^).

## Results

**Figure 2:**
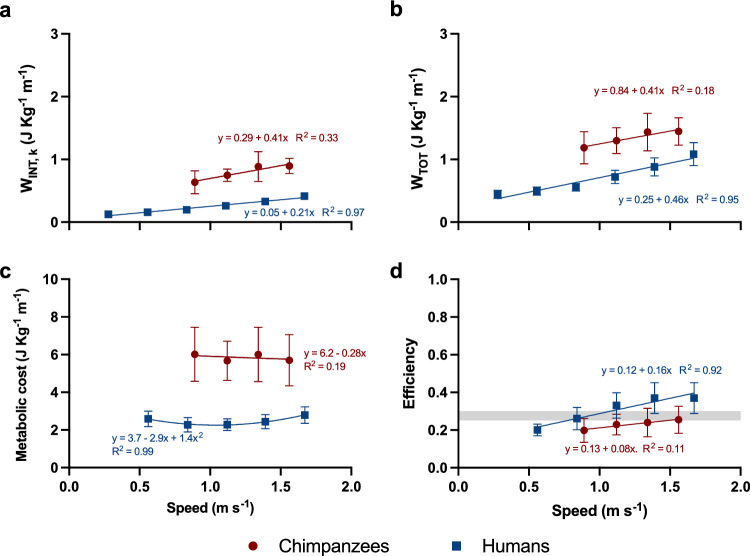
Mechanical work, metabolic cost, and efficiency. Internal kinetic mechanical work (W_INT,k_), total mechanical work (W_TOT_), metabolic cost, and locomotor efficiency are plotted as a function of speed. Data from Pavei et al.^29^ for humans. Error bars: standard deviation. Solid lines: regression lines for chimpanzees (red) and humans (blue). Shaded area in panel (**d**): maximum efficiency range for isolated muscles contracting concentrically^43^.

Compared with chimpanzees, humans have lower stride frequency and duty factor at all speeds, and a lower *q’* (Fig. 1, Table 2), leading to lower W_INT,k_ (Fig. 2). In the common speed range 1.1–1.4 m s^−1^, W_EXT_ ranges from 0.46 to 0.55 J kg^−1^ m^−1^ for humans and averages 0.55 J kg^−1^ m^−1^ for chimpanzees. Because of concomitantly decreased W_INT,k_ and W_EXT_, humans walk with less W_TOT_ than chimpanzees (Fig. 2, Supplementary Fig. S4). As values of C from humans are proportionally lower than those of chimpanzees at all speeds, between-species differences in efficiency are smaller than differences in either C or W_TOT_ (Fig. 2, Supplementary Fig. S4).

## Discussion

In this paper, we provide evidence that humans walk bipedally with less mechanical internal work than chimpanzees. Total mechanical work is also lower in humans than in chimpanzees, making between-species differences in efficiency smaller than those in metabolic cost.

### Mechanical work

**Figure 3:**
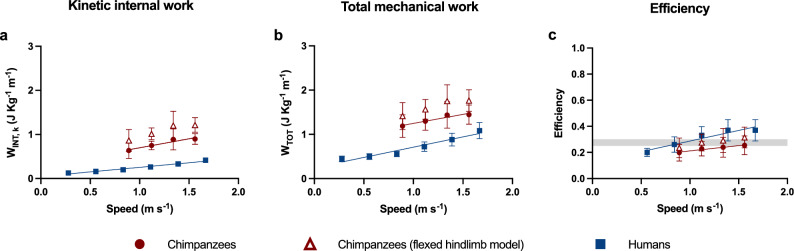
Mechanical work and efficiency assuming a flexed hindlimb. In addition to the data presented in Fig. 2, this plot shows how assuming a flexed lower limb for chimpanzees impacts modeled W_INT,k_, W_TOT_, and efficiency. In the flexed limb model, a mean knee flexion angle of 125° (with 180° representing knee full extension) and a mean angle of the foot relative to the vertical of 80° was considered (see Supplementary Material S1). Error bars: standard deviation.

At a given speed, W_INT,k_ is proportional to three terms: stride frequency, a monotonous function of duty factor, and an ‘inertial term’ that lumps relative limb lengths and masses distribution^28^ (Eq. 1). Such a model is coherent with stereophotogrammetric calculations of W_INT,k_^22,44^, and explains the mechanisms driving changes in W_INT,k_ between and within species^28,29,45^; however, it assumes equal relative gyration radii and center of mass position for all limbs. As limb mass distribution differs between chimpanzees and humans, we generalized such model to avoid these assumptions (Eqs. 10 and 11). The model also assumes fully extended limbs, but Supplementary Material S1 and Fig. 3 show that limb flexion would not relevantly alter calculations of mechanical work and efficiency. In the range of speeds between 0.45 and 1.67 m s^−1^, humans walk with a lower stride frequency^2,29^, contributing to a 22–25% reduction in estimated W_INT,k_ (Table 2, Fig. 1); humans also have a lower duty factor at low speeds (which further reduces W_INT,k_ by up to 49%), but this difference diminishes at higher speeds (Table 2, Supplementary Fig. S4). Even if the human upper limb has a greater relative gyration radius than chimpanzees’ forelimb, this is compensated by its lower fractional mass and length (Table 2)^23,24^; altogether, this reduces *q’*, and hence W_INT,k_ by an additional 16%. As a result, humans have a ~ 60% lower W_INT,k_ than chimpanzees. These different strategies may reflect distinct optimization goals in the two species: a higher duty factor and stride frequency may optimize safety and stability in chimpanzees, while lowering them curbs the mechanical demands of walking in humans; greater distal masses in the upper limbs favor climbing and brachiation, while shifting them proximally and to the lower limbs reduces the cost of walking^46^.

Besides W_INT,k_, work is done to overcome joint friction during locomotion (W_INT,f_)^33^. Generalizing its formula, W_INT,f_ is proportional to *β*_*U*_*/R*_*U*_^*2*^ + *β*_*L*_*/R*_*L*_^*2*^, where *β*_*U*_*, β*_*L*_*, R*_*U,*_* R*_*L*_ are the damping coefficients (N m s rad^−1^) and length (m) of the upper and lower limbs, respectively (Supplementary Material S2). If human damping coefficients *β*_U_ and *β*_L_ are taken from Minetti et al.^33^ and the same are assumed for chimpanzees, humans would do less W_INT,f_ because of the concomitantly increased R_U_ and R_L_. However, this assumption is challenged by the interspecies differences in soft tissue distribution and anatomy of the proximal limb joints^47^, potentially causing great differences in damping coefficients. Therefore, W_INT,f_ was not quantified here or included in W_TOT_; this quantity however should not be negligible, and once data on damping become available, estimates of mechanical work in chimpanzees could be improved.

Finally, the interplay between W_EXT_ and W_INT,k_ is not solved yet: summing them could be considered an “upper bound” estimate of whole-body mechanical work^48,49^ and their metabolic correlate may seem counterintuitive since C of human walking increases when people are not allowed to swing their arms^50^. However, the fact that the *net* effect of removing upper limb swing increases C does not imply that limb swing happens at no metabolic cost. On the contrary, muscle blood flow measurements in animal and modeling studies^51,52^, the existence of dissipation between and within joints^33^ and the fact that W_INT,f_ values in humans are of the same magnitude as those of W_INT,k_ themselves^33^ challenge the idea that limb swing can happen at negligible cost and that calculations of limb swing costs can be ignored. Further models should also include the effect of natural limb oscillation frequency^48,53,54^ and W_INT,f_^33^ on C.

### Locomotor efficiency

Due to the lower W_EXT_^6^ and W_INT,k_, humans had a lower W_TOT_: consequently, the disparities in locomotor efficiency between the two species were considerably smaller than those in C (Fig. 2). While this suggests that a portion of the lower C in humans can be attributed to reduced mechanical work, the extant differences in efficiency between the two species hint that mechanical work does not explain all variations in C. Moreover, efficiency was speed-dependent (Fig. 2); for chimpanzees, this was due to the fact that W_EXT_ and C were approximately constant, while W_INT,k_ increased with speed. Finally, differences in W_TOT_ are less pronounced when comparisons are done at dynamically similar speeds (Supplementary Fig. S4).

Locomotor efficiency can also be expressed as the product of muscle efficiency and transmission efficiency^55^, and humans may have optimized both components. Muscle efficiency may be enhanced due to optimized muscle architecture and a higher proportion of type I fibers^1,4,56^; it also increases when muscles operate at advantageous velocities^43,57,58^, but data are lacking for chimpanzees walking. On the other hand, transmission efficiency increases when elastic energy is stored and released in the tendons and connective tissues of the hip, ankle, and foot^59–64^; this can result in overall (“apparent”) efficiency being higher than that of isolated muscle (Fig. 2). Such a hypothesis is supported by observations by O’Neill and colleagues^19^ who found that humans, but not chimpanzees, can save a relevant fraction of mechanical work during a stride through elastic mechanisms; this could account for some of the remaining between-species differences in efficiency in Fig. 2. When using mechanical work data from O’Neill and colleagues^19^ to compute locomotor efficiency, we found values of 0.23 for chimpanzees and 0.37 for humans walking at 1.09 m s^−1^ (Supplementary Material S5). O'Neill et al.^19^ also estimated how much work humans could save due to elastic mechanisms: by subtracting it from total mechanical work, a “muscle” efficiency of 0.25 is derived. At the same speed, our efficiency estimates are 0.22 for chimpanzees and 0.29 for humans (Supplementary Material S5). This suggests numerical consistency between the present results and those from O’Neill and colleagues^19^ and that the remaining discrepancies in locomotor efficiency between species can be attributed to factors not captured by mechanical work calculations, including optimized muscle–tendon mechanics in humans. Transmission efficiency also improves when muscles operate at advantageous lengths and moment arms, and with reduced lower limb co-contractions^55^: both mechanisms may contribute to reducing C in humans thanks to their ability to walk with more extended hips and knees^1,65^. In contrast, the pelvis orientation in chimpanzees forces them to keep these joints bent during the stance phase^3,14,65^, likely at the cost of increased isometric contraction of lower limb muscles. This can increase C without affecting W_EXT_. Transmission efficiency also depends on belly and tendon gearing^66^ and soft tissue deformations^19,67^; further studies are needed to elucidate their role in the comparative physiology of walking.

### Limitations and future perspectives

This work relies on published data to estimate differences in W_INT,k,_ between humans and chimpanzees and generate hypotheses on how they affect the cost of walking. The present is an analytical estimate of W_INT,k_: the model can yield reasonable estimates since it holds for a range of gaits, speeds, and species^28,44,45^, but experiments are needed to measure W_INT,k_ in chimpanzees and test these hypotheses by collecting mechanical and metabolic data on the same participants. Experimental measures would also show whether mediolateral movements, which are neglected in this model but are potentially relevant for chimpanzees, affect internal work calculations. Of note, experimental data on W_EXT_ and C come from adult chimpanzees with heterogeneous age and biometry (Table 1); however, chimpanzees’ walking mechanics does not relevantly change after the age of 5 years^68^.

On one hand, further experiments are required to measure quantities that could refine estimates of mechanical work in chimpanzees, including the precise amount of external work done during the double support phase^69,70^, the mechanical work actually performed at the muscle level^71,72^, and tendon elastic storage and recoil, which would require combined ultrasound and kinetic data^59^. On the other hand, between-species differences in metabolic cost have also been addressed by force-based rather than work-based models^3,53,73^; future work may elucidate whether these two contributions are mutually exclusive, additive^74^ or equivalent^75^.

## Conclusions

Compared to chimpanzees, the lower cost of human walking is associated with a combined reduction in the work to accelerate and raise their body center of mass and the work to swing their limbs. When both terms are considered, estimated walking efficiency is still higher in humans than chimpanzees, suggesting that factors beyond mechanical work also contribute to such differences in metabolic cost between the two species.

### Supplementary Information

Supplementary Information.

## Data Availability

No new data was generated for this study.

## List of symbols

a: Proximal distance of the lower limb center of mass as a fraction of limb length
b: Upper limb length as a fraction of lower limb length
C: Metabolic cost
d: Duty factor
EqO_2_: Energy equivalent of oxygen
*Fr*: Froude number
*g*: Gravity acceleration
*m*: Body mass
m'L: mass of the lower limb as fraction of body mass
m'U: mass of the upper limb as fraction of body mass
*q'*: Inertial factor
R: Average length of the four limbs
R_L_: Lower limb (hindlimb) length
R_U_: Upper limb (forelimb) length
SF: Stride frequency
*v*: Average progression speed
*V̇O*_*2rest*_: Oxygen uptake at rest
*V̇O*_*2ss*_: Oxygen uptake at steady state
*W*_*EXT*_: External mechanical work
*W*_*INT,f*_: Internal frictional mechanical work
*W*_*INT,k*_: Internal kinetic mechanical work
W_TOT_: Total mechanical work
*β*: Damping coefficient
*β*_L_: Sum of the damping coefficients for the lower limb (hindlimb)
*β*_U_: Sum of the damping coefficients for the upper limb (forelimb)
γ: Limb radius of gyration as a fraction of limb length
γ_L_: Radius of gyration of the lower limb (hindlimb) as a fraction of limb length
γ_U_: Radius of gyration of the upper limb (forelimb) as a fraction of limb length
