# Supplementary material for: The work to swing limbs in humans versus chimpanzees and its relation to the metabolic cost of walking
Source: Sci Rep. 2024 Apr 18;14:8970. doi: 10.1038/s41598-024-59171-8 (PMC11026468; doi:10.1038/s41598-024-59171-8)
Supplement: Supplementary file 1 — Supplementary Information. [file 41598_2024_59171_MOESM1_ESM.docx]

The work to swing limbs in humans versus chimpanzees and its relation to the metabolic cost of walking.

Francesco Luciano*^1^, Luca Ruggiero*^1,2^, Alberto E. Minetti^1^ and Gaspare Pavei^1^

SUPPLEMENTARY MATERIALS

**S1. Validity of the estimates of internal kinetic mechanical work**

In the present study, the estimated W_INT,k_ for chimpanzees is compared with experimental values for humans. Would these results hold if human values were derived using the same modeling approach? If W_INT,k_ is estimated from the data provided by Pavei and colleagues^1^, it aligns closely with its experimental measurements (Supplementary Figure 1). Thus, the utilization of a modeled W_INT,k_ *per se* does not appear to bias the comparisons between chimpanzees and humans.

**Supplementary Figure 1.** Comparison between measured and modeled W_INT,k_ for human bipedal walking. Calculated from Pavei et al.^1^. Dashed line: identity line.

However, such comparisons could still be biased by specific assumptions inherent to the W_INT,k_ model. Minetti’s model^2^ assumes equal angular excursion between the upper limb (or forelimb) and lower limb (or hindlimb); such an assumption was not originally tested for chimpanzees but seems reasonable in light of kinematic data collected on such species. For instance, Finestone et al.^3^ found that chimpanzees walk at their freely chosen speed with a retraction angle of 24 [23 ; 28] degrees (mean [Q1; Q3]) for the forelimb and 27 [24; 31] for the hindlimb, and a protraction angle of 34 [30; 35] degrees for the forelimb and 31 [28; 34] degrees for the hindlimb.

Furthermore, the q’ term is calculated under the assumption of fully extended limbs. While this is reasonable for humans, it may not hold for chimpanzees, who exhibit greater knee flexion angles during walking. For context, from O’Neill et al.^4^ we can calculate a mean knee flexion angle of 27 ± 21° for humans and 55 ± 18° for chimpanzees during a stride (0° representing full knee extension). Here we estimate the magnitude and direction of such bias in the W_INT,k_ model. To simplify this analysis, we consider a lower limb that touches the ground at the tip of the foot T and whose knee and ankle are bent throughout the stride with constant angles β and δ, respectively (Supplementary Figure 2).

**Supplementary Figure 2.** Geometric model of the lower limb. The positions of the hip (H), knee (K), and ankle (A) joints are depicted on the parasagittal angle, while T indicates the position of the tip of the foot. *l_T_, l_S_*, and *l_F_:* length of the thigh, shank, and foot segments, respectively.

With the reference frame set at the hip joint, the position of *T* is:

$\boldsymbol{T}: \left( \begin{matrix} T_{x} \\ T_{y} \end{matrix} \right)=\left( \begin{matrix} l_{F}\sin\left( \delta\right) \\ -\sqrt{{(l}_{T}^{2}+l_{S}^{2}+2l_{T}l_{S} cos(\beta))-l_{F}\cos\left( \delta\right)} \end{matrix} \right)$ (S1)

where *l_T_*, *l_S_*, and *l_F_* are the lengths of the thigh, shank, and foot segments, and the Cosine Rule is applied for the y coordinates. The lower limb length (L_LL_) is:

$L_{LL}=\sqrt{{T_{x}}^{2}+{T_{y}}^{2}}$ (S2)

Defining the distances of the centre of mass for each segment relative to the proximal extremity as RP_T_, RP_S_, and RP_F_ for the thigh, shank, and foot, respectively, the positions of CM_T_, CM_S_, and CM_F_ are:

$\boldsymbol{CM}_{\boldsymbol{T}} : \left( \begin{matrix} CM_{T_{x}} \\ CM_{T_{y}} \end{matrix} \right)=\left( \begin{matrix} {RP}_{T} \sin\left( \alpha\right) \\ -{RP}_{T} \cos\left( \alpha\right) \end{matrix} \right)$(S3)

$\boldsymbol{CM}_{\boldsymbol{S}} : \left( \begin{matrix} CM_{s_{x}} \\ CM_{s_{y}} \end{matrix} \right)=\left( \begin{matrix} l_{T}\sin\left( \alpha\right)- {RP}_{S} \cos\left( \beta+\alpha-\frac{\pi}{2} \right) \\ - l_{T}\cos\left( \alpha\right)- {RP}_{S} \sin\left( \beta+\alpha-\frac{\pi}{2} \right) \end{matrix} \right)$(S4)

$\boldsymbol{CM}_{\boldsymbol{F}} : \left( \begin{matrix} CM_{F_{x}} \\ CM_{F_{y}} \end{matrix} \right)=\left( \begin{matrix} {RP}_{F} \sin\left( \delta\right) \\ -\sqrt{\left[ l_{T}^{2}+l_{S}^{2}-2l_{T}l_{S} cos(\beta) \right]}- {RP}_{F} \cos\left( \delta\right) \end{matrix} \right)$ (S5)

where *sin(α)* and *cos(α)* are given by the Sine Rule and the Pythagorean trigonometric identity:

$\sin\left( \alpha\right)=\frac{l_{S}}{AH}\sin\left( \beta\right)= \frac{l_{S}}{-\sqrt{\left[ l_{T}^{2}+l_{S}^{2}-2l_{T}l_{S} cos(\beta) \right]}}\sin\left( \beta\right)$ (S6)

$\cos\left( \alpha\right)=\sqrt{1-\sin^{2} \left( \alpha\right)}$ (S7)

Given *m_T_*, *l_T_*, *CM_T_* the mass, length, and centre of mass for the thigh, *m_S_*, *l_S_*, and *CM_S_* for the shank, and *m_S_*, *l_S_*, and *CM_S_* for the foot, the position of the centre of mass of the lower limb *CM_LL_* and its distance from the hip joint (*r*) are:

$\boldsymbol{CM}_{\boldsymbol{LL}}=\left( \frac{1}{m_{LL}} \right)\left[ m_{T}\boldsymbol{CM}_{T}+m_{S}\boldsymbol{CM}_{S}+m_{F}\boldsymbol{CM}_{\boldsymbol{F}} \right]$ (S8)

$r=\sqrt{{{CM}_{LL_{x}}}^{2}+{{CM}_{LL_{y}}}^{2}}$ (S9)

The moment of inertia of each segment within the limb relative to their centre of mass *I_TCoM_*, *I_SCoM_* and *I_FCoM_* is given by:

$I_{TCoM}= m_{T} {g_{T}}^{2}$ (S10)

$I_{SCoM}= m_{S} {g_{S}}^{2}$ (S11)

$I_{FCoM}= m_{F} {g_{F}}^{2}$ (S12)

where *g_T_*, *g_S_* and *g_F_* are the radii of gyration of the lower limb segments relative to their centre of mass. The moment of inertia of the lower limb relative to its centre of mass *I_LLCoM_* is hence:

$I_{LLCoM}= I_{TCoM}+\left( m_{T} {\bar{{CM}_{T}{CM}_{LL}}}^{2} \right)+I_{SCoM}+\left( m_{S} {\bar{{CM}_{S}{CM}_{LL}}}^{2} \right)+I_{FCoM}+\left( m_{F} {\bar{{CM}_{F}{CM}_{LL}}}^{2} \right)$ (S13)

where $\bar{{CM}_{T}{CM}_{LL}}$ , $\bar{{CM}_{S}{CM}_{LL}}$ and $\bar{{CM}_{F}{CM}_{LL}}$ are the distances between the centre of mass of the thigh, shank, and foot, and the centre of mass of the lower limb (according to the Parallel Axis Theorem). The equations above can also be applied to the upper limb to determine the position of the centre of mass *CM_UL_*, length *L_UL_*, and moment of inertia relative to the centre of mass *I_ULCoM_*. In this special case, however, it can be assumed that α = β = δ = 0. Finally, *L*, *a* and *g* can be differentially calculated for the extended lower limb (L_LLE_, a_LLE_, g_LLE_), flexed lower limb (L_LLF_, a_LLF_, g_LLF_), and extended upper limb (L_ULE_, a_ULE_, g_ULE_) and W_INT,k_ can be recalculated by generalizing Equation 9 as:

$W_{INT,k}=SF v_{ST}\left( 1+\left( \frac{d}{1-d} \right)^{2} \right)\frac{\pi^{2}}{4}\left[ \left( {m'}_{L}{a_{LL_{E}}}^{2} \right)+\left( {m'}_{U}{a_{UL_{E}}}^{2}\frac{{L_{UL_{E}}}^{2}}{{L_{LL_{E}}}^{2}} \right)+\left( {m'}_{L}\frac{{g_{{LL}_{F}}}^{2}}{{L_{LL_{F}}}^{2}} \right)+\left( {m'}_{U}\frac{{g_{UL_{E}}}^{2}}{{L_{LL_{E}}}^{2}} \right) \right]$ (S14)

This equation can be solved to produce the values illustrated in Figure 3. Inertial parameters were taken from Druelle et al.^5^ and Winter^6^, with a mean value of β of 125° (180°-55°), and of δ of 81° from O’Neill et al.^4^. Accounting for flexed lower limb is expected to increase W_INT,k_. Such increase occurs because on one side hindlimb gyration radius decreases in chimpanzees, but on the other side the decreased hindlimb length increases at a given speed and stride frequency the angular acceleration and the rotational kinetic energy requirements, with almost constant translational work. This would also cause a slight increase in W_TOT_ and efficiency for chimpanzees: discrepancies in efficiency between species would be slightly lower, and a higher proportion of the differences in metabolic cost would be explained by variations in mechanical work. In conclusion, lower limb flexion may account for some of the remaining variations in efficiency observed between humans and chimpanzees in Figure 2; however, its impact is relatively small compared with the disparities in metabolic cost and mechanical work between the two species. The assumption of a straight lower limb gives a simple and conservative estimate of mechanical work and is therefore used in the main text for the sake of clarity.

**S2. Model for W_INT,f_ with different damping and length for upper and lower limbs**

In Minetti et al.^7^ the mechanical cost of transport to overcome the internal friction of a single limb (C_mif_, J m^-1^) and the mass-specific mechanical cost to overcome the internal friction of all four limbs (C_mifa_ or W_INT_,_f_, J kg^-1^ m^-1^) were given by:

$C_{mif}=\frac{\pi^{2}\beta}{8 R_{L}^{2}}v$(S15)

$C_{mifa}=W_{INT, f}= \frac{\pi^{2}B}{8 m R^{2}}v$(S16)

with

$B= \sum_{i=1}^{4} \beta_{i}$(S17)

where *β_i_* are the damping coefficients (N m s rad^-1^) of each proximal limb joint. This equation assumes that the four limbs have the same length *R*. However, this assumption may limit comparisons across species with different upper limb (forelimb) versus lower limb (hindlimb) length ratios. Hence, C_mifa_ can be alternatively expressed as the sum of the cost to overcome the internal frictions of the two upper limbs (or forelimbs) C_mifU_ and the cost to overcome the internal frictions of the two lower limbs (or hindlimbs) C_mifL_:

$C_{mifa}=W_{INT, f}= \frac{1}{m}\left( C_{mifU}+C_{mifL} \right)=\frac{\pi^{2} \beta_{U} v}{m 8 R_{U}^{2}}+\frac{\pi^{2} \beta_{L} v}{m 8 R_{L}^{2}}=\frac{\pi^{2}}{8 m}v\left( \frac{\beta_{U}}{R_{U}^{2}}+\frac{\beta_{L}}{R_{L}^{2}} \right)$(S18)

where *β_U_* is the damping coefficient of the upper limbs under unloaded swinging, and *β_L_* is the damping coefficient of the lower limbs given by the sum of their tensile unloaded and compressive loaded damping coefficients^7^. Damping coefficients have been experimentally determined for humans by Minetti et al.^7^; however, their values in chimpanzees are unknown.

**S3. Model selection for external mechanical work**

To assess the relation between W_EXT_ and speed in chimpanzees, data were fitted with zero-, first-, and second-order mixed effects models. Their Akaike Information Criterions (AIC) were -79.1, -75.0, and -75.5, respectively; hence, the zero-order model was chosen. The distribution of its residuals was checked and reported below (Supplementary Figure 3).

**Supplementary Figure 3**. Quantile-quantile plot for the observed and expected residuals for the zero-order mixed effects model. The plot and the model checks, included the Kolmogorov-Smirnov (KS) test, the dispersion test, and the outlier test were obtained through the “DHARMa” package in R.

**S4. Comparisons of cost, mechanical work, and efficiency at dynamically equivalent speeds**

In the main text, mechanical and metabolic variables are compared at absolute speeds; here, they are compared at dynamically equivalent speeds expressed as Froude numbers^8,9^ (Supplementary Figure 4):

$Fr=\frac{v^{2}}{g l}$(S19)

where *v* is the average progression speed (m s^-1^), *g* is the gravity acceleration on Earth (9.81 m s^-2^) and *l* is the lower limb or hindlimb length (m; chimpanzees: 0.46 ± 0.05 m; humans: 0.90 ± 0.03 m; mean ± standard deviation).

**Supplementary Figure 4.** *Mechanical work, metabolic cost, and efficiency***.** Internal kinetic mechanical work (W_INT,k_), total mechanical work (W_TOT_), metabolic cost, and locomotor efficiency are plotted as a function of Froude number (Fr). Data from Pavei et al.^1^ for humans. Error bars: standard deviation. Solid lines: linear regression for chimpanzees (red) and humans (blue). Shaded area in panel d: maximum efficiency range for isolated muscles contracting concentrically^10^.

**S5. Evidence from comparative studies on joint work**

In a recent study, O’Neill and colleagues^11^ compared the dimensionless total mechanical work of the lower limb of three humans and three chimpanzees walking bipedally. This measure differs from the whole-body W_TOT_ calculated in the present paper for two main reasons: first, the total mechanical work from O’Neill and colleagues is given by the summed contributions of the hip, knee, and ankle joint work instead of the summed W_EXT_ and W_INT,k_; second, it was made dimensionless by dividing by *m g l*, where *m* is the body mass, *g* is the gravity acceleration, and *l* is the lower limb or hindlimb length. This term will hence be referred to as ω_TOT_JW_. The corresponding dimensional total mechanical work in J kg^-1^ m^-1^ can be calculated as:

$W_{TOT\_JW}=\omega_{TOT\_ JW} g l SF v^{-1}$(S20)

where *SF* is the stride frequency (Hz) and *v* is the average progression speed (m s^-1^). At a speed of 1.09 m s^-1^, ω_TOT_JW_ was 0.27 for chimpanzees and 0.12 for humans. At that speed, a metabolic cost (C) of 5.9 J kg^-1^ m^-1^ is expected for chimpanzees and 2.20 J kg^-1^ m^-1^ for humans. If efficiency is then calculated as $W_{TOT\_JW}C^{-1}$, this would be equal to 0.23 for chimpanzees and 0.37 for humans. O'Neill et al.^11^ also estimated how much work humans could save due to elastic storage and release at the tendons and connective tissues of the hip and ankle: by subtracting it from ω_TOT_JW_, this led to a total ‘muscle fiber’ work of 0.08, which would correspond to an efficiency of 0.25. At the same speed, our estimates of efficiency are 0.22 and 0.29 for chimpanzees and humans, respectively. This suggests that the results from our paper are coherent with those from O’Neill and colleagues^11^; it also suggests that the remaining differences in walking efficiency between species can be accounted for by optimized muscle-tendon mechanics in humans.

**Supplementary references**

1. Pavei, G., Biancardi, C. M. and Minetti, A. E. (2015). Skipping vs. running as the bipedal gait of choice in hypogravity. *J Appl Physiol* 119, 93–100.
2. **Minetti, A. E.** (1998). A model equation for the prediction of mechanical internal work of terrestrial locomotion. *J Biomech* **31**, 463–468.
3. Finestone, E. M., Brown, M. H., Ross, S. R. and Pontzer, H. (2018). Great ape walking kinematics: Implications for hominoid evolution. *Am J Phys Anthropol* 166, 43–55.
4. O’Neill, M. C., Lee, L.-F., Demes, B., Thompson, N. E., Larson, S. G., Stern, J. T. and Umberger, B. R. (2015). Three-dimensional kinematics of the pelvis and hind limbs in chimpanzee (Pan troglodytes) and human bipedal walking. *J Hum Evol* 86, 32–42.
5. Druelle, F., Schoonaert, K., Aerts, P., Nauwelaerts, S., Stevens, J. M. G. and D’Août, K. (2018). Segmental morphometrics of bonobos (Pan paniscus): are they really different from chimpanzees (Pan troglodytes)? *Journal of Anatomy* 233, 843–853.
6. Winter, D. A. (1979). Biomechanics of human movement. Wiley.
7. Minetti, A. E., Moorhead, A. P. and Pavei, G. (2020). Frictional internal work of damped limbs oscillation in human locomotion. *Proc Biol Sci* 287, 20201410.
8. Alexander, R. M. (1976). Estimates of speeds of dinosaurs. *Nature* 261, 129–130.
9. Alexander, R. M. (1984). Stride length and speed for adults, children, and fossil hominids. *Am J Phys Anthropol* 63, 23–27.
10. Smith, N. P., Barclay, C. J. and Loiselle, D. S. (2005). The efficiency of muscle contraction. *Prog Biophys Mol Biol* 88, 1–58.
11. O’Neill, M. C., Demes, B., Thompson, N. E., Larson, S. G., Stern, J. T. and Umberger, B. R. (2022). Adaptations for bipedal walking: Musculoskeletal structure and three-dimensional joint mechanics of humans and bipedal chimpanzees (Pan troglodytes). *J Hum Evol* 168, 103195.
